# Review of a Lecture upon Clastic Anatomical Models

**Published:** 1869-06

**Authors:** 


### AMERICAN JOURNAL
OF
### DENTAL SCIENCE.

Vol. III. THIRD SERIES--JUNE, 1869. No. 2.

### ARTICLE I.

### *Review of a Lecture upon Clastic Anatomical Models.*

Delivered before the Baltimore College of Dental Surgery, by F. G. Lemercier Co-operator of Dr. Auzoux and Professor of the Polytechnic Association of Paris.

The functions of nerves and ganglia are so totally different that we cannot mistake the action of either.

The nerves are for conduction only.

The ganglia are for reception--transmission--concentration and diffusion; and in a yet higher form of ganglia, we have the registering of impressions and the elaboration of these registered impressions—or the process of reasoning. Registering of the impressions by the ganglia is the 1st step and the resurrecting of these registered impressions is the 2nd step in the process;—we call this, taken as a whole, *memory.*

We would call attention here to the fact that ganglia *never originate* impressions; that some agent to act upon them through the nerves, as in the cases of common sensation and special sensations; or some agent to act directly upon the ganglia, as we believe the soul acts upon the cerebral ganglia, or cerebral hemispheres, is an absolute necessity. Light for the eye; sound for the ear; odors for the nose; sapid substances for the tongue; and in common, general or tactile sensibility, we have contact, pressure, variation of temperature &c.; upon the cerebral hemispheres some agent must act, or else there is a break in the unity of the nervous system; that agent is the soul.

### *The Spinal Cord.*

We are compelled to make a repetition in order that the functions of the cerebro-spinal axis, or spinal cord and brain may be studied as a whole.

The cord was shown to consist of a double series of ganglia commissured on the median line; and these ganglia were so closely packed in longitudinal series, that there was a blending of anatomical structure to such an extent that the individuality of the ganglia was lost, and they formed an extension of gray cineritious matter, continued on through the cord into the cranial cavity, and into the *intracranial* portions of the *cord*, or what we term the *Medulla Oblongata.*

This ganglionic extension was more or less central, being completely enveloped by layers of white cords or nerves, which thoroughly covered it in and concealed it from our sight.

An examination of these nerves proved that the posterior half were afferent or sensory, and the anterior half were efferent or motor; that closer examination showed that many of the nerves coming from the periphery, (*i. e.* Afferent) terminate in this ganglionic matter; and many others originating in the ganglionic matter, passed out to the periphery, (*i. e.* Efferent.)

That other nerves (probably the posterior half of the cord) were continued on to the brain, and thus carried impressions from the periphery to the central ganglia.

That the anterior half of the cord, evidently contained many nerves which had their origin in the brain or intracranial ganglia, and were the *tracts* for impressions from these ganglia to the exterior of the body, or periphery.

Now the nerves (called afferent) which begin in the periphery and terminate in the ganglionic matter of the cord, and those nerves (called efferent) which begin in this ganglionic matter of the cord and terminate in the periphery; constitute with their special mass of ganglionic matter, *a complete nervous arc—a complete physico-reflex apparatus*, capable of acting independently of the brain. And this apparatus corresponded in anatomy and physiology with a ganglion and its nerves as seen in the ganglionic cord, of the class *articulata.* That the anterior and posterior portions of the cord, and in fact all of the cord which ran to or descends from the brain, [and was not ganglionic,] was simply commissural, and connected or commisured segments of the cord with each other, and each segment and all the segments with the intracranial ganglia.

### *The Functions of the Spinal Cord.*

1st. Physico-reflex—In virtue of its ganglia and nerves.

2nd. Commissural.—In virtue of its nerves, (anterior and posterior.)

Comparative Anatomy teaches this; Pathology confirms it; Vivisection verifies it.

The "Magnified Encephalon" was next in order and the lecturer proceeded to dissect it, piece by piece, or ganglion by ganglion. He showed the minute anatomy of every portion, making a desultory commentary upon its supposed functions at the same time. Here however we prefer giving our own opinions, &c.

### *The Medulla Oblongata.*

This is the intracranial portion of the spinal cord, and somewhat larger than the cord, being also more complex in its anatomy. The cord has a median fissure in front and behind, or anterior and posterior, and two lateral fissures; this gives two symmetrical halves and each half divided into three columns:—an anterior, posterior and a column between the two, or a median column.

Now the Medulla Oblongata has three lateral fissures instead of two, and therefore four columns in each half instead of three; of these four columns, the one anteriorly and the one posteriorly, lying by the anterior and posterior median fissures, are called anterior and posterior pyramids and are exactly opposite to each other; the column next to the anterior pyramid is called the Olivary Tract or Body—the column next to the posterior pyramid is called the Restiform Tract or Body; the olivary and restiform tracts lie side by side and between the anterior and posterior pyramids.

These columns are continuous with the cord below, but their relations to the ganglia above were, and have been for many years, a vexed question.

Now if the anatomy of this model be correct, and we certainly endorse it so far as our knowledge at present extends, then we would say that Drs. Auzoux and Lemercier have contributed a most exquisitely beautiful, and a most admirably adapted specimen for the study of the Encephalon and its ganglia.

We shall endeavor to give concisely this anatomy and then give the functions of these ganglia. Posteriorly the two pyramids, wheel right and left out of position and leave a triangular space called the *Calamus Scriptorus;* the Restiform bodies by about one ,half of their columns, reinforce these wheeling columns from the pyramids, and the two blend as one and immediately form one of the "crura cerebelli," and run into the lateral hemispheres of the cerebellum; anteriorly, the anterior pyramids send a winding band around the olivary body and over this body, which under the name of "arciform fibres," winding back, seeks the crura cerebelli and blends with them upon each side; each crura cerebelli has therefore fibres from 1st. Posterior Pyramids—2nd. Restiform tract—3rd. Anterior Pyramids; it contains therefore both motor and sensory elements.

Now there remains to be accounted for—1st. part of the Restiform—2nd. Olivary tract, and 3rd. the remainder of the anterior Pyramids; these run beneath the broad transverse band of white fibres known as the Pons Varolii, and emerging beyond form the diverging "crura cerebri."

Now the cerebellum is built up, as it were, upon the crura cerebelli; the broad band of white fibres stretching across the front of the "crura cerebri" at their origin, runs into the right and left hemispheres of the cerebellum, and terminates in a rounded constricted cord; now these constricted terminations of the "Pons Varolii" are called also "crura cerebelli." So far, we have two sets of crura cerebelli.

*4th Ventricle.*

As the posterior pyramids have wheeled out right and left and turned upwards to form the "crura cerebelli," and as about one-half of the restiform column has joined in this movement out, there must arise two things; 1st, the triangle, or calamus scriptorus; 2nd, the uncovered central ganglionic matter of the cord comes into view lying upon this triangular space, now also, as only one-half of the restiform tract wheels out to become "crura cerebelli," and as the other one-half passes on forward to become "crura cerebri," and converges towards its fellow opposite, we have necessarily a lozenge shaped space; formed by the diverging pyramids below and the converging halves of the restiform above.

This space is beneath the cerebellum;—behind and resting upon the posterior aspects of the anterior pyramids and olivary bodies, and between the diverging posterior pyramids and the converging halves of the restiform tracts above; this is the 4th ventricle; and upon its floor the ganglionic matter comes into view, and here the 8th pair of nerves, according to Soemmering, have their ganglia and their origin; in the Carp, a species of fish, the auditory ganglia is a distinct ganglion upon the floor of the 4th ventricle. The half (upon each side) of the restiform body which runs up and blends with "crura cerebri," terminates partially in the "testis" of that side, and hence has been called "processus e cerebello ad testes"; the halves approximate but do not unite along their entire depth and a small bridge of nervous matter is thrown between them; this of course covers up the space and makes a canal, this canal is called the "aqueduct of Sylvius;" or "iter e tertio ad quartum ventriculum."

The Tubercula Quadrigemina, called also the Nates and Testes, rest upon this bridge and upon the lateral restiform tracts: but here the restiform tract has lost its name, and asssumed that of the *posterior surface of the crura cerebri*, hence the Tubercula Quadrigemina are said to be situated upon the posterior surface of the crura cerebri, and above the aqueduct of Sylvius.

*The Crura Cerebri.*

Have an anterior or motor portion derived from the anterior pyramids and olivary bodies, and posterior derived from the restiform, or from that half of the restiform which did not go up into the cerebellum and become "crura cerebelli;" this posterior portion has fibres which come from the cerebellum intrinsically, and therefore, though the restiform gave a portion of itself to the cerebellum, that organ returned the gift in a series of fibres that help to make up the "processus e cerebello ad testes."

Now the question arises what becomes of the Crura Cerebri?

These crura ascending, diverge somewhat and terminate abruptly in two large ganglia; the posterior portions in the Optic Thalami and the anterior portions in the Corpora Striata. These ganglia lie upon the floor of the lateral ventricles;—optic thalami parallel and not in contact, having an open space called the 3rd ventricle between them; the corpora striata, anterior to the optic thalami, are not parallel but convergent and pyriform, the larger ends meeting on the median line; the smaller ends divergent and permitting the optic thalami to come up between them. The optic thalami receives the sensory and the corpora striata receives the motor tract.

The model of Dr. Lemercier showed all of this, but it showed more;— it showed that the fibres which ran into the optic thalami *terminated in it*; and other fibres took their *origin* in the optic thalami and diverging widely as they emerged, were distributed in every direction ascending towards the superficies of the hemispheres, or towards the cerebral ganglia and *terminated in the gray convolutions of these ganglia.* Again, that many other fibres *began* in these *gray convolutions,* in every part of these hemispheres, converging descended and passed into the *corpora striata* and there *terminated*; that other fibres *began* in the *corpora striata* and ran down as the anterior columns or motor tract of the crura cerebri, and of the spinal cord.

In the statement of the facts as they really exist we should say that, the posterior portions of the "crura cerebri," run into the optic thalami and stop, for these are their proper ganglia; that fibres arise in the optic thalami and run out towards the the cerebral convolutions and terminate in these convolutions; that fibres begin in these convolutions and run converging down to the corpora striata and there terminate; and that fibres begin in the corpora striata and running down become the anterior portions of crura cerebri and cord.

Now it will be seen, that, as the anterior or motor tract is *descending* from the encephalon, the statement made about the "arciform fibres" coming from the medulla oblongata and running into the crura cerebelli and finally into the cerebellum, must be exactly reversed; these arciform fibres begin in the cerebellum and passing down converge into narrow cords on the crura cerebelli, and winding around the olivary body they descend blended with the anterior, and seek a terminal distribution with the anterior or motor tract of the cord.

Mr Solly of England (in his admirable work upon the Structure, Physiology and Diseases of the Brain, pages 188, 189, 190) teaches that these fibres descending from the cerebellum to join the medullary oblongata, are composed of three or four distinct sets, superficial and deep, and thinks that they form a very large element of the descending motor tract of the cord.

Certainly the physiology of the cerebellum requires exactly this anatomical arrangement; and we cordially endorse Mr. Solly; and Dr. Lemercier's illustration of Solly's and Magendie's views, throws a flood of light upon this organ as well as upon the cerebrum and other ganglia.

*The Functions of the Cranial Ganglia.*

The Medulla Oblongata being the intra cranial portion of the " spinal cord " has the two functions of the cord, that is physico-reflex action and commissural action; and we find many sensory and motor cranial nerves arising from it, as the 3rd. 4th. 5th. 6th. and 7th. pairs, and also the 9th. 10th. and 12th. pairs; but it has also some special ganglia which pre-over special functions as

1st. The ganglia of the Olivary body—for Deglutition.

2nd. The ganglia of the Restiform—for Respiration.

*Pons Varolii*

Is a commissure for the two cerebellar hemispheres and contains a ganglion called " tuber annulare," which is thought to be connected with semi-consciousness and semi-volition, but this is not as yet demonstrated as true.

*Cerebellum*

Is for (1) Muscular co-ordination. (2) Muscular Sense. By muscular sense we mean the faculty of interrogating the muscular system and ascertaining its exact condition and the exact amount of force which we can elicit for any given purpose; by muscular co-ordination we mean the combining and directing of the action of many muscles to the attaining of one end,—one common definite result. Muscular co-ordination means *concentrated unity* of action.

There are many able men, (as Trousseau of Paris for example) who do not comprehend or do not believe in the existence of this " Muscular Sense;" they ask, how will you distinguish it from " Muscular Co-ordination ?"

The distinction to us is an obvious and an easy one, and can be illustrated very readily

The "*Inspector*" of a division in an army has nothing to do with the movements of troops; his duty is to ascertain the "*exact fighting*" condition of every company, regiment, and brigade— he reports this information to the proper officer (Division Commander), and upon his report, this officer *acts*, knowing exactly how much fighting power he can wield ; now suppose the action to begin, we will at once see him co-ordinating the movements of the brigades ; unitizing their different actions for one result, and avoiding in every possible way irregular and desultory action,—here the one head commanding co-ordinates the efforts of severalbrigades into one common effort of the division, and, if he does his duty, he obtains "*unity of action—unity of result.*"

The *Inspector* corresponds to the muscular sense. The *Division Commander* to the co-ordinate power. The muscular sense deals with the muscular system in its passive state ; the co-ordination deals with it in its active state.

A close examination of this model, showed a general commissuring or interchange of fibres between the different cranial ganglia. The optic ganglia, called Tubercular Quadrigemina, were commissured with the cerebellum and cerebrum ; the cerebral hemispheres or cerebral ganglia were commissured with each other by the great transverse commissure, the Corpus Calosum and the Fornix blended ; and by the crura cerebri were commissured with all ganglia below them ; *but*, and here comes in a remarkably nice point, only through the " optic thalami."

"All roads lead to Rome," all Sensory nerves lead directly or indirectly to the optic thalami ; afferent nerves (called sensory) may terminate in the ganglia of the cord, but unless the impressions conveyed to the cord are transmitted to the brain and become sensations, these nerves are not sensory ; they are afferent and physico-reflex only.

All sensory nerves end either in special ganglia, or end in the optic thalami ; and those ending in special ganglia, as the auditory, optic, &c., are commissured with the optic thalami, by cords from ganglia to ganglia. All motor nerves *begin* in the corpora striata, except the arciform fibres *descending* from the cerebellum.

From the anatomy of the optic thalami and the corpora striata, we perceive that they bear a relation to each other exactly parallel with that of the posterior and anterior gray horns of the cineritious, or ganglionic matter of the cord, *i.e.* sensation and motion.

### *The Optic Thalami.*

These are the last *grand registering and receiving depots;* they are the *common centres* of all impressions; they receive and register all sensations, special or general.

Now the fibres radiating out from these ganglia towards the superficies of the brain, must be divided into two classes, 1st, those conveying to the cerebral ganglia information of what has been registered; 2nd, those fibres through which the *will* acts in resurrecting or bringing out these impressions, in the process of "re-collection;" and this word *recollection* is most appropriate and most happily true. Memory is but the effort by which we collect again, or re-collect these impressions registered in the optic thalami.

All impressions are here received and stored up, registered,—they remain as long as life lasts, each one modifying every other and being itself modified by what has gone before and by that which comes after. It is true then that "first impressions are lasting"? yes, and also modifying and controlling for good or evil.

### *The Corpora Striata.*

Great Motorial Centres—and all impressions descending from the brain,— that is from the cerebral ganglia, and having for their object voluntary muscular action, must pass into the Corpora Striata; and from these descends the proper motor influence. The "motor influence" does not descend really from the cerebral ganglia, but an order, as it were, is transmitted to the corpora striata and these ganglia send out the "*motor impulse.*" The corpora striata under orders from the cerebral ganglia, evolve motor impulses.

### *Sensori-reflex.*

These acts are performed often involuntarily though consciously, we are conscious of the sensation, *i.e.* the registered impression, but the will does not act to evolve the motor impulse, only the optic thalami and corpora striata act, the cerebral ganglia are quiescent.

*Cerebral Ganglia.*

Seat of the Intellect;—the Ganglia of Soul; the intelligence and powers of acquisition being almost directly as the number, size, weight and complexity of the convolutions. We believe the cerebral ganglia to be the *material instrument* of the *immaterial agent* or essence which is called the *Soul.*

It has been impossible to do justice to this lecture by mere statements, the models were everything as regards clearness and interest. We have omitted a good deal and will conclude with a statement of ganglia and functions, as far as our present knowledge extends.

*Intra Cranial Ganglia.*

(1) Ganglia of the olivary bodies—Lingual and Deglutition. (2) Ganglia of the Restiform—Respiration. (3) Ganglia of the 4th Ventricle—Auditory. (4)Ganglia of the Pons Varolii—Semi-consciousness. (5) Ganglia of the Cerebellum—Co-ordination and Muscular Sense. (6) Ganglia of the Crura Cerebri—Oculo motor. (7) Corpora Quadrigemina—Optic Ganglia, vision. (8) Optic Thalami—Touch, common Sensibility and Grand Register. (9) Corpora Striata—General Motor. (10) Olfactory Bulbs—Sense of Smell. (11) Cerebral Hemispheres—Seats of the Intellect.

The study of the anatomy of the nervous system by these models from comparative anatomy and the magnified encephalon, presents one of the most beautiful and most instructive fields open to the investigation of the student. The accuracy of anatomical details, the variety of the models, and the skill with which the series have been arranged, should command the highest praise and most grateful acknowledgements from the profession generally.

